# Bilateral Suprascapular Nerve Cryoneurolysis for Pain Associated With Glenohumeral Osteoarthritis: A Case Report

**DOI:** 10.1016/j.arrct.2023.100256

**Published:** 2023-01-25

**Authors:** Fraser MacRae, Eve Boissonnault, Mahdis Hashemi, Paul Winston

**Affiliations:** a Western University, School of Physical Therapy, London, Canada; b Vancouver Island Health Authority, Victoria, Canada; c Division of Physical Medicine and Rehabilitation, Centre Hospitalier de l'Université de Montréal, Montréal, Canada; d Division of Physical Medicine and Rehabilitation, University of British Columbia, Faculty of Medicine, Vancouver, Canada

**Keywords:** Nerve block, Osteoarthritis, Pain, Rehabilitation

## Abstract

Osteoarthritis is a leading cause of disability, typically treated with exercise, analgesics, injections, or surgeries. Cryoneurolysis is an established technique for the treatment of pain, including osteoarthritis that may provide an alternative for patients in whom surgery is not appropriate and conservative measures have failed. We present our experience with a 78-year-old man with severe pain from bilateral glenohumeral osteoarthritis. Their condition is complicated by several concurrent diagnoses, leaving them ineligible for surgical intervention, despite pharmacologic treatments proving insufficient to manage their pain. As an alternative, bilateral cryoneurolysis of the suprascapular nerve was performed at the suprascapular notch. Pain and disability scores both lessened on the Brief Pain Inventory Score, Disabilities of the Arm Shoulder and Hand (change of 9 points after 170 days) as well as the Shoulder Pain and Disability Index (change of 19 points after 170 days). The patient had improved active and passive range of motion for flexion, abduction, and external rotation of the shoulder. Improvements endured to follow-up at 170 days. There were no negative side effects as a result of the procedure.

Osteoarthritis is a painful condition caused by a combination of biomechanical and metabolic features resulting in changes in articular cartilage and bone.^1^ Affecting 17% of the population of Japan over 40 years old, an estimated 24 million Americans, and anticipated 6.5 million Canadians by 2031, osteoarthritis is a leading cause of disability worldwide.^2^, ^3^, ^4^ Glenohumeral osteoarthritis (GHOA) is less common than osteoarthritis of the weight bearing joints but is a painful condition that can severely affect independence by limiting pain-free range of motion about the shoulder.^5^ While pharmaceutical treatments are available, severe GHOA requires a total shoulder arthroplasty for complete resolution as there is no known cure. Arthroplasty is invasive and requires a long recovery period. Wait times as well as the patient's ability to tolerate the procedure can be problematic; in Canada, many patients wait over a year for their appointment.^6^ Alternative interventions are required to improve the quality of life of patients on wait lists, and to treat pain for patients who are not surgical candidates.

Cryoneurolysis is a procedure where clinicians percutaneously freeze sensory or motor nerves using a specialized probe capable of rapidly freezing to -88° C. The nerve responsible for the propagation of the pain signal is identified and selectively lysed. When the nerve is exposed to the extreme cold secondary axonotmesis occurs. Courtesy of the constant supply of warm blood, surrounding tissues are unaffected by the procedure.

Most of the sensory innervation to the shoulder is provided by the suprascapular nerve which also innervates the supraspinatus and infraspinatus muscles.^7^ By targeting the nerve at the suprascapular notch, it does not affect the supraspinatus muscle but will weaken the infraspinatus. The lateral pectoral nerve, the axillary nerve, and the lower subscapular nerve also contribute articular branches to the shoulder.^8^ All of these other nerves also provide motor innervation to muscles of the pectoral girdle including the pectoralis major, pectoralis minor, deltoid, and subscapularis, among others. By breaking down the axon of the suprascapular nerve with cryoneurolysis, pain transmission is effectively blocked. As such, cryoneurolysis does not directly address the cause of the pain and does not resolve the pathophysiology of GHOA but instead interrupts the pain signal, potentially leading to immediate and lasting relief. Institutional ethics approval was not required for this report. Informed consent was obtained for the purpose of this case report in accordance with the Case Report Guidelines (CARE).

## Case

A 78-year-old man is referred to an outpatient physical medicine and rehabilitation clinic with debilitating bilateral shoulder pain from GHOA. The pain dates back 2 years and seems to worsen every day. The patient reports that their pain has been significantly worse over the past 2 months and describes it as a 3/10 constant ache, elevating to 10/10 with forward flexion or abduction of the arm at the shoulder. The pain affects his ability to perform daily hygiene and dressing. The patient presented as an inoperable candidate with a complex history, including porocarcinoma and squamous cell carcinoma, atopic dermatitis, carotid disease, lumbar stenosis, chronic obstructive pulmonary disease, asthma, osteoarthritis of the cervical and lumbar spine, and osteopenia. The patient completed radiation therapy for their cancer 2 months prior to presentation at the clinic.

**Fig 1:**
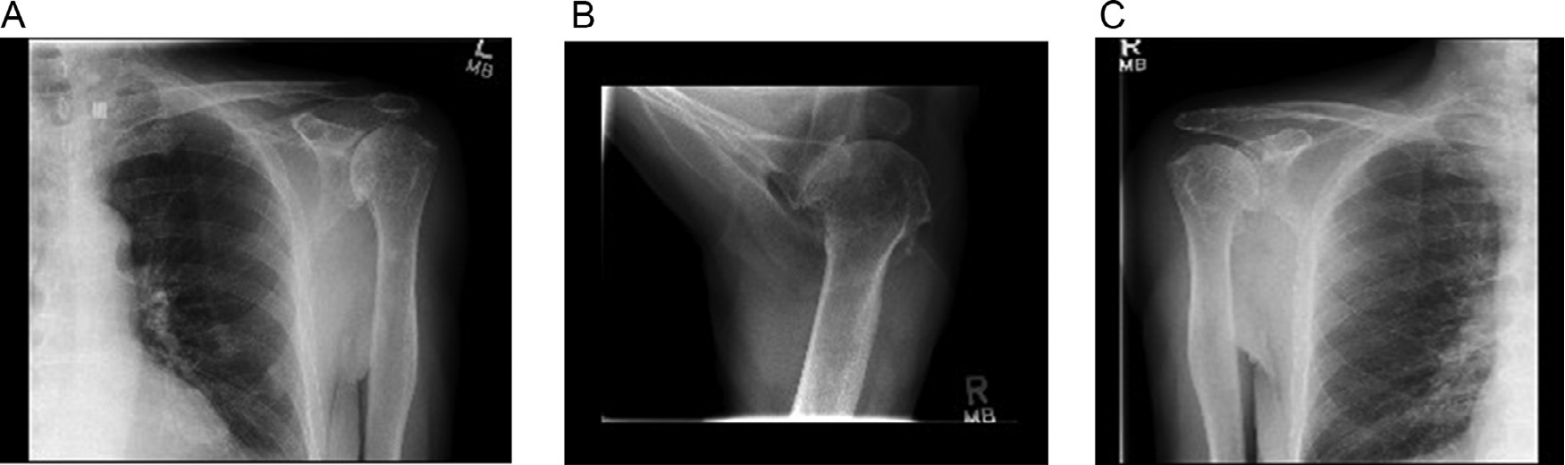
Bilateral shoulder radiographs obtained 1 month before cryoneurolysis show right sided moderately severe degenerative change and left moderate to severe arthritic change with flattening of the humeral head and prominent inferior osteophytes. (A) Frontal plane view of the left shoulder. (B) Axillary view of the right shoulder. (C) Frontal plane view of the right shoulder.

The patient had shoulder radiographs 1 month prior to presentation at the clinic that revealed right sided moderately severe degenerative change in the glenohumeral joint and severe joint space loss (fig 1). Views of the left shoulder demonstrated moderate to severe arthritic change with flattening of the humeral head and prominent inferior osteophytes. The patient has tried several nonpharmacologic therapies including physiotherapy, exercise, and massage therapy with little effect. The patient has tried topical analgesia including diclofenac cream but this was quickly discontinued because of complications with their history of skin cancer. 1-2 daily tablets of acetaminophen 650 mg provided some inadequate relief.

After a detailed explanation of the possible side effects including local and neurogenic pain, bruising, hematoma, and rare chance of blood vessel puncture, the patient consented to bilateral cryoneurolysis of the suprascapular nerve. Baseline testing was taken on the Brief Pain Inventory Score, Disabilities of the Arm Shoulder and Hand (DASH), and Shoulder Pain and Disability Index (SPADI), as well as active and passive range of motion and grip strength and the Box and Block test.

**Fig 2:**
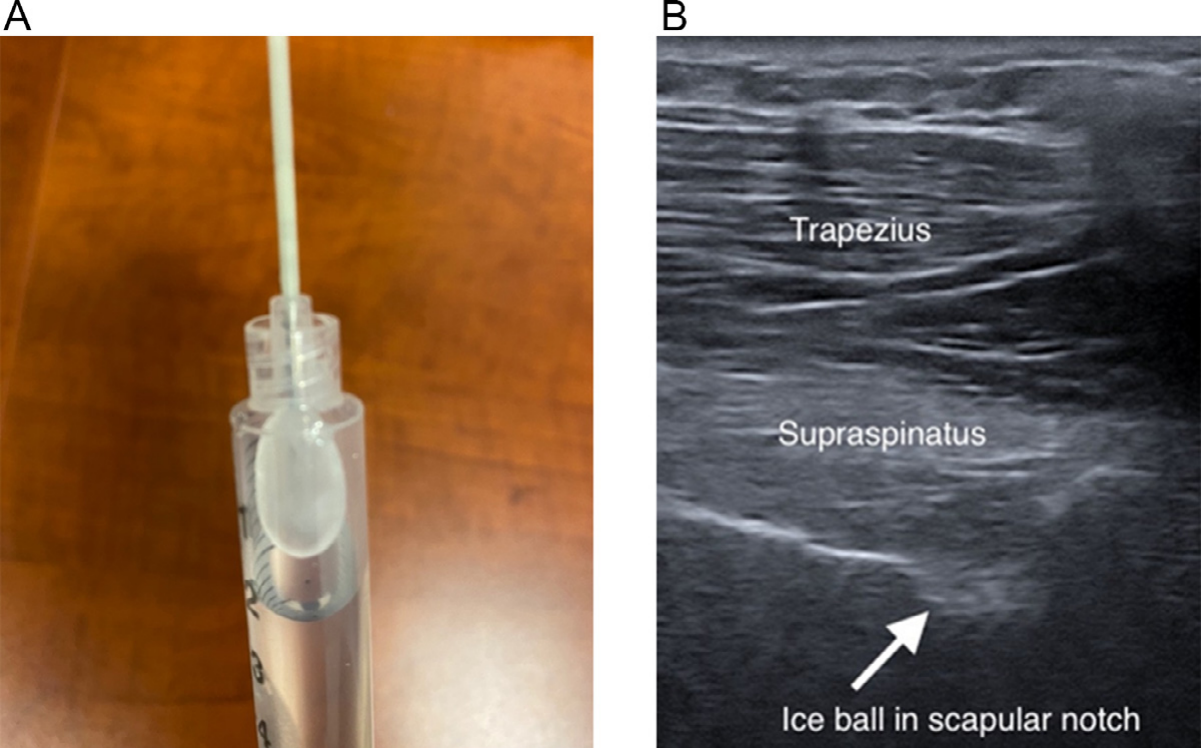
Equipment and perioperative ultrasonographic images of cryoneurolysis. (A) iovera system 190 Smart Tip, iovera, Pacira, NJ, USA, with ice ball created from water inside a syringe. (B) Fully formed ice ball during cryoneurolysis, visualized in the suprascapular notch with ultrasound.

The patient was prepared with 4% chlorhexidine gel to clean the skin. The skin in the region of the injection was infiltrated with 1 cc of 1% lidocaine without epinephrine. A 16-gauge angiocath was inserted into the skin to facilitate insertion and avoid frostbite. Under ultrasound guidance, the handheld probe^a^ (fig 2) was placed in close proximity to the suprascapular nerve at the suprascapular notch.^9^ Two adjacent lesions, each lasting 106 seconds, were performed to ensure complete nerve disruption. After the second lesion, the patient was already noticing the analgesic effects of the procedure and consented to have the procedure for the other side. The shoulder range of motion and pain were improved in the minutes after the procedure, and the patient was able to dress himself much more easily to leave the clinic.

## Results

Follow-up at 6 weeks, the patient reported that his function had improved. His wife noted that he made breakfast for the first time today and was able to perform some other tasks much more easily. He still experienced significant pain when he moved the arm excessively. Tolerable passive range of motion improved in all directions with the most significant changes in external rotation (50° in right side and 35° in left side). Also, significant improvement in external rotation active range of motion was noted (15 and 25 in right and left side). The minimum clinically important difference for glenohumeral range of motion is 14°-24°.^10^ At 7 months, he noted that he could do almost any required personal task if performed slowly. His range of motion and function and pain scores had all improved. Complete details of the range of motion changes, patient reported score on the DASH, Brief Pain Inventory. SPADI, Box and Block, and grip strength are reported in tables 1 and 2.

**Table 1:** Shoulder range of motion in flexion, abduction, and external rotation

	Baseline				40 Days				170 Days				
	Active		Passive		Active		Passive		Active		Passive		
	Rt	Lt	Rt	Lt	Rt	Lt	Rt	Lt	Rt	Lt		Rt	Lt
**Shoulder flexion (degrees)**	90	120	95	125	95	110	125	135	100	125		115	135
**Shoulder abduction (degrees)**	90	80	110	115	85	105	115	110	80	110		120	120
**Shoulder external rotation (degrees)**	35	20	35	35	50	45	85	70	75	55		85	75

**Table 2:** Function, pain, and grip strength at baseline, 6 weeks, and 7 months

		Baseline		40 Days		170 Days	
		Rt	Lt	Rt	LT	Rt	Lt
**Box and Block test**		48	36	50	35	50	38
**Grip strength (kg)**		20	18	18	18	20	20
**Dash score**		59.48		62		50	
**SPADI score**	**Pain score**	74		58		50	
	**Disability score**	82.5		77.5		61	
	**Total score**	78.4		70		59.6	

## Discussion

A 78-year-old patient presented with severe GHOA, complicated by several other concurrent diagnoses. The patient was not a surgical candidate, and alternative management strategies have not been sufficient to address the pain. Cryoneurolysis of the suprascapular nerve was performed bilaterally. After the procedure, the patient's function was preserved, as evidenced by consistent scores on the Box and Block test and grip strength measurement. Pain and disability scores both lessened—indicative of reduced pain and increased ability (table 2). There was clinically significant improvement on the SPADI of more than 13 points. For the DASH, the minimally detectable change at 95% confidence interval has been shown to be between 8 and 17 (mean 13) by the Institute for Work Health.^11^ It is notable that the patient's baseline DASH score of 59 is considered very high.^12^ Active and passive range of motion improved for both sides for shoulder flexion, abduction, and external rotation (table 1). All changes persisted or improved up to 170 days post-cryoneurolysis.

Medical frailty prevents many patients with osteoarthritis from being eligible for arthroplasty. With over 50 years of studied efficacy and safety, cryoneurolysis is a minimally invasive procedure with the potential to address pain from osteoarthritis.^13^ Though the use of cryoneurolysis for inoperable glenohumeral disease has not been explored from a functional perspective, there have been case studies targeting the suprascapular nerve in the peri-surgical period. Percutaneous cryoneurolysis was performed preoperatively to alleviate postoperative pain after shoulder rotator cuff repair in 2 patients.^9^ Both patients had postoperative analgesia with pain scores consistently <2 on a 0-10 numeric rating scale and required significantly less opioid for a decreased time relative to historical controls.

Neuroablation via pulsed radiofrequency is a technique that has also shown promise for the management of pain from osteoarthritis.^14^ Pulsed radiofrequency ablation uses a specialized hot probe to target sensory nerves. There are studies assessing the use of pulsed radiofrequency of the suprascapular nerve for shoulder disorders. Cristiani and Hernandez described a pediatric septic arthritis case with osteophytic transformation of the glenohumeral joint.^15^ Similar to our case, the patient had relief at 6 months of follow-up. Compared with pulsed radiofrequency, cryoneurolysis has the advantage that a small portable handheld machine can be used in any setting; at the bedside, outpatient clinic, pre- or post-operative, or in long term care. This is especially advantageous for patients with limited mobility.

Intra-articular corticosteroid injections are a commonly employed non-surgical management strategy for GHOA. There is limited evidence that documents the reliability of this technique.^16^^,^^17^ Recent studies have demonstrated the effectiveness of this technique.^18^^,^^19^ Metzger et al found that after a single injection, patients with GHOA had clinically and statistically significant reductions in pain lasting 4 months.^19^ Corticosteroid injections require repeat injections every few months and have both local and systemic risk factors.

## Limitations

The presented results are from a sample size of n =1 which greatly limits the generalizability of the findings. As the patient had little active and passive range of motion at baseline, it is possible that any weakness of the infraspinatus caused by cryoneurolysis was undetected. Larger, randomized trials are necessary to fully understand the potential uses of cryoneurolysis for pain from GHOA.

## Conclusions

Our patient presented with debilitating shoulder pain and loss of function due to GHOA. A complicated medical history negating the possibility for surgery. The patient had immediate and sustained reductions in pain, reduced disability scores, and improved tolerated passive range of motion at 7 months of follow-up. Cryoneurolysis of the suprascapular nerve could be considered to manage pain from GHOA if less invasive alternatives are not providing sufficient relief; if surgery wait times are lengthy, or if patients are not surgical candidates. The handheld cryoneurolysis system can be used in any outpatient setting as there is no console or wires. With surgical wait times further delayed because of the COVID-19 pandemic, novel interventions should be explored.

## Suppliers

a. Iovera ststem 190 smart tip; iovera

## Appendix Supplementary materials

Image, video 1
